# Therapeutic Potential of Chinese Medicine for Endogenous Neurogenesis: A Promising Candidate for Stroke Treatment

**DOI:** 10.3390/ph16050706

**Published:** 2023-05-07

**Authors:** Lin Li, Xiao Li, Rui Han, Meirong Wu, Yaolei Ma, Yuzhao Chen, Han Zhang, Yue Li

**Affiliations:** 1 Institute of Traditional Chinese Medicine, Tianjin University of Traditional Chinese Medicine, Tianjin 301617, China; 2 Key Laboratory of Pharmacology of Traditional Chinese Medical Formulae, Tianjin University of Traditional Chinese Medicine, Ministry of Education, Tianjin 301617, China; 3 State Key Laboratory of Component-Based Chinese Medicine, Tianjin University of Traditional Chinese Medicine, Tianjin 301617, China

**Keywords:** stroke, neurogenesis, neural stem cells, Chinese medicine

## Abstract

Strokes are a leading cause of morbidity and mortality in adults worldwide. Extensive preclinical studies have shown that neural-stem-cell-based treatments have great therapeutic potential for stroke. Several studies have confirmed that the effective components of traditional Chinese medicine can protect and maintain the survival, proliferation, and differentiation of endogenous neural stem cells through different targets and mechanisms. Therefore, the use of Chinese medicines to activate and promote endogenous nerve regeneration and repair is a potential treatment option for stroke patients. Here, we summarize the current knowledge regarding neural stem cell strategies for ischemic strokes and the potential effects of these Chinese medicines on neuronal regeneration.

## 1. Introduction

Strokes are the leading cause of long-term disability and the second leading cause of mortality and morbidity worldwide [1]. Over the past decade, many neuroprotective drug candidates for acute ischemic stroke that showed promise in experimental animal stroke models have failed in human clinical trials [2]. Recombinant tissue plasminogen activator (rtPA) and thrombectomy are unique FDA-approved therapies for acute ischemic strokes [3]. However, these treatments have several limitations. They have a narrow temporal window (within 4.5 h of stroke onset), and the risk of hemorrhagic conversion restricts the application of rtPA [4]. Statistically, only approximately 2–4% of stroke patients can garner a benefit [3]. Although current stroke treatment strategies in the subacute and chronic phases, such as rehabilitation, the long-term management of antithrombotic therapy (antiplatelet and anticoagulation), and the treatment of traditional risk factors, have shown remarkable efficacy, the overall outcome remains poor [5,6,7,8,9].

Neurological disorders have a complex underlying molecular pathogenesis involving irreversible brain injury and neuronal repair [5]. Neurogenesis is critical for brain development and damage repair [6]. Enhanced neurogenesis has been observed in hypoxic neural stem cells (NSCs) in vitro and ischemic brains in vivo, including in neonatal mice, adult rats, and aged humans [10,11,12]. However, this category of spontaneous neurogenesis does not recover neurological function in patients who have had an ischemic stroke because most NSCs cannot survive to induce differentiation owing to tissue ischemia and reperfusion injury [13]. A continuous search for more potent alternative therapeutic approaches for neurogenesis has been conducted [14]. Recently, stem cell therapy has attracted considerable interest in regeneration therapy owing to its satisfactory preclinical and clinical results [15,16,17,18].

Among all stem-cell-based treatments for stroke, the transplantation of exogenous progenitor/stem cells and the activation/recruitment of endogenous NSCs are promising strategies for neuronal regeneration. However, many unresolved fundamental problems hamper the clinical application of NSC transplantation [19,20]. In contrast, in endogenous therapeutic strategies, NSCs in the adult brain are induced to proliferate, migrate toward the damaged area, differentiate into different cell types to contribute to functional repair, and become functionally active and integrated into the surrounding tissue to enhance neurological recovery [21]. Pharmacological interventions related to promoting adult endogenous neurogenesis may be a more practical approach to stroke treatment without the difficulties and challenges faced by exogenous stem cell transplantation therapy [22,23].

Multiple systemic factors and factors associated with pathogenesis, such as age, vascular risk, duration of therapy, and the site or extent of central nervous system (CNS) damage, may affect the recovery or repair of neurological deficits after a stroke. The development of brain circuits and recovery of neurological function after a stroke require a complex series of coordinated neurodevelopmental events [23]. Adult neurogenesis and neural regeneration after ischemic injury are not only related to NPC proliferation but also include neuronal migration, synaptic neogenesis, neural loop reconstruction, axonal regeneration, and neurovascular unit remodeling. There may be differences in the repair capacity of neurological functions in different types of cerebral ischemic injury, and such discrepancies can be attributed in part to the complexity of the CNS response after a stroke, differences in synaptic plasticity, and limitations in axonal regeneration [24]. Synaptic plasticity during the development of neural loops determines the possibility of integrating newborn neurons into the neural loops [25]. Axonal regeneration is the main requirement for the functional remodeling and restoration of the lost neural circuit, which depends on various neurobiological changes, such as the extent of neuronal impairment, myelin formation, synaptogenesis, astrocytic scars, and chondroitin sulfate proteoglycan levels [26,27]. Therefore, these differences lead to different prognoses in patients with different types of strokes.

Chinese medicines, which include single compounds, herbal extracts, and formulations, have become potential therapies for stroke treatment and are widely utilized clinically in China and other Asian countries owing to their multiple pharmacological actions, including anti-inflammatory, antioxidant, and antiapoptotic effects, thereby presenting many opportunities for the exploration of new TCM-derived drugs for this disease [28]. Furthermore, recent studies have suggested that certain Chinese medicines potentially affect endogenous neural regeneration after a stroke, implying that they may provide a prolonged window for stroke treatment [29]. In this review, we summarize the current knowledge regarding NSC therapy for ischemic brain injury and the underlying effects of these Chinese medicines on endogenous neuronal regeneration.

### Endogenous Neurogenesis

Most human organs and tissues contain stem or progenitor cells. These stem cells coordinate tissue homeostasis and repair by generating new cellular units that respond to physiological or pathological conditions. However, the brain has long been considered an exception. The original idea was that the generation of newborn neurons and neurogenesis occur only during embryonic development and that neuronal death in the adult CNS could only be replaced by the proliferation of glial cells [30]. NSCs were first isolated from the striatum and hippocampus of adult mice in 1992 [31,32] and are defined as pluripotent cells in the CNS that can self-renew and differentiate into neurons, astrocytes, and oligodendrocytes [33]. Furthermore, recent extensive studies have shown that adult mammalian pluripotent stem cells in the brain also self-proliferate and differentiate into neurons and glial cells [34]. These cells not only self-renew or proliferate but also migrate [35,36].

It is currently accepted that the subgranular zone (SGZ) of the dentate gyrus (DG) of the hippocampus and the subventricular zone (SVZ) adjacent to the lateral ventricle are the main areas in which adult neurogenesis occurs [37]. The NSCs residing in these zones have the potential to enhance the regeneration of damaged tissue by replacing lost or old cells through developmental mechanisms. In addition, NSCs have been found in areas of the brain and peripheral nervous system, such as the neocortex, adult olfactory bulb, striatum, and spinal cord [38,39,40]. The necessary processes in adult neurogenesis include the proliferation, differentiation, and fate determination of NSCs and progenitor cells when they undergo symmetric and asymmetric division, migration, changes in plasticity, and the integration of newborn neurons into existing neural circuits. The distribution of endogenous NSCs in different regions of the brain and the factors that influence neurogenesis play important roles in endogenous neurogenesis (reviewed in detail in [38]).

Most endogenous NSCs in the adult brain reside in a resting state. Under normal physiological conditions, a stimulating external environment, appropriate physiological activities (e.g., running and studying), growth factors, and oxygen concentration can promote the activation, proliferation, migration, and differentiation of resting-state NSCs. In addition, specific cytokines, such as stromal cell-derived factor 1 (SDF-1) and angiopoietin-1, are produced by damaged tissues in the brains of adult animals to induce the activation of endogenous NSCs during disease states, such as cerebral ischemia, epilepsy, or trauma [41]. The aberrant activation of NSCs leads to proliferation at ectopic and orthotopic sites and migration, differentiation, and integration at the site of injury (such as the cortex and striatum) to repopulate damaged brain tissue [42]. However, spontaneous neurogenesis has been suggested to be inefficient and inadequate because of the lack of sufficient, survivable, and high-quality neurons [43]. In addition to this mechanism of NSC activation, a population of radial glial cells located in the lateral ventricular SVZ, also called B cells, are in a state of quiescence or slow proliferation [44]. B cells proliferate to form rapidly dividing neural progenitors (C cells), while C cells proliferate vigorously and differentiate into neuroblasts (also known as A cells). These neuroblasts migrate along the rostral migration stream (RMS) to the olfactory bulb to participate in the regeneration and differentiation of neurons in the middle of the olfactory nerve regeneration and differentiation neurons [45]. In contrast, endogenous NSCs localized in the SGZ migrate directionally into the stratum granulosum, mature into neurons, and integrate into hippocampal circuits [46]. As a result, new excitatory neurons are generated in the DG that play a role in learning, memory, and cognitive functions [47]. In addition, reactive astrocytes are widely distributed in the neocortex, cerebellum, striatum, amygdala, substantia nigra, hypothalamus, and spinal cord and are potential sources of progenitor cells [48]. Reactive astrocytes also exhibit NSC-like properties [49].

## 2. Endogenous Neurogenesis Mechanism: An Approach to Restoring Neurological Function

**Figure 1:**
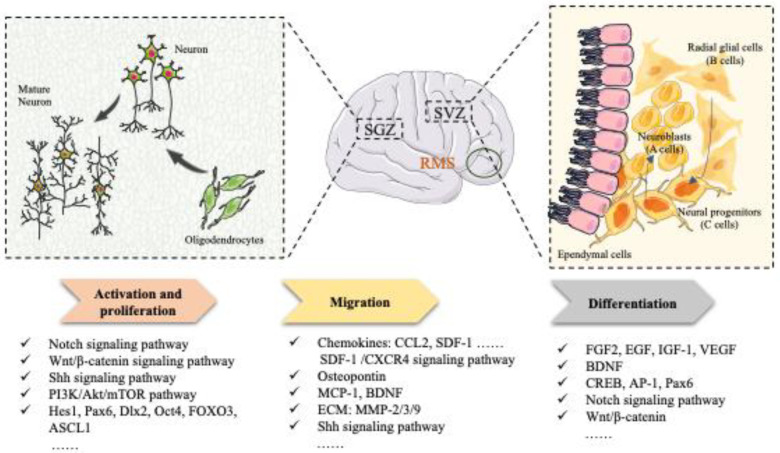
The general process of endogenous neurogenesis.

**Figure 2:**
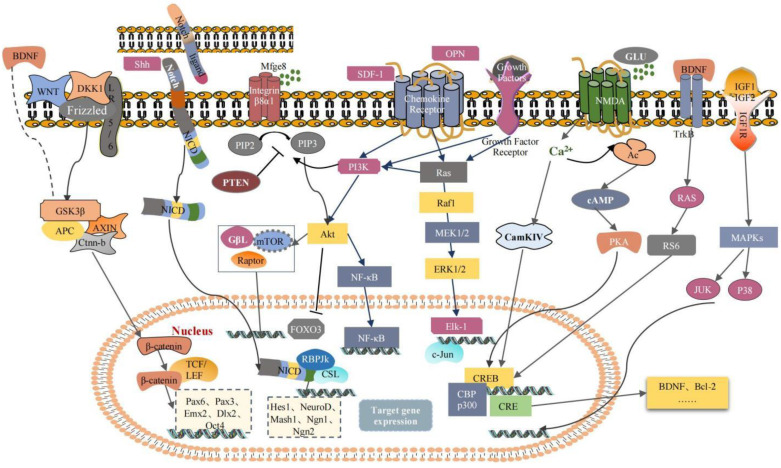
Molecular mechanisms in the regulation of endogenous neurogenesis after stroke.

Stroke leads to brain tissue necrosis, nerve cell death, and a loss of order in the intricate wiring of neurons, glial cells, and the vascular system [21]. Catastrophic changes in the cellular microenvironment caused by injury to the blood–brain barrier, excitotoxicity, mitochondrial dysfunction, oxidative stress, and neuroinflammation after stroke can affect the survival, differentiation, and neurogenesis of endogenous NSCs [50]. Increasing evidence has supported that ischemic stroke induces neurogenesis in multiple brain regions [51,52]. Newborn neurons must proliferate, migrate, differentiate, and integrate to play a functional role in recovery after a stroke [53]. The basic mechanisms underlying the neuroprotective effects of NSCs in strokes have been extensively demonstrated, including nutritional support, the attenuation of inflammatory responses, immunomodulatory functions, the targeted replacement of neurons, the re-creation of neural circuits, and the restoration of neural tissues that were damaged [54,55]. Therefore, improving endogenous neurogenesis mechanisms to replace lost neurons and promote tissue repair in the CNS has long been an exciting therapeutic target for strokes. However, we must recognize that endogenous neurogenesis after a stroke is not a unitary or one-step process. It is a range of progressive events that involves the activation and proliferation of NSCs, the migration of these cells to damaged areas, the differentiation of progenitor cells into different lineages, and the integration of neurological functions (Figure 1 and Figure 2). In this process, the Wnt/β-catenin, Notch, and other signaling pathways affect endogenous neurogenesis by regulating the expression of related proteins, playing a neuroprotective role. Therefore, therapies corresponding to each of these steps may be beneficial for brain functional recovery and tissue repair after a stroke.

### 2.1. Activation and Proliferation

The proliferation of NSCs is greatly increased in both the SVZ and SGZ 2–5 days after a stroke, peaks on days 7–8 after ischemia, and continues for approximately 30 days [56,57]. By analyzing brain specimens from patients who had a stroke, it was also confirmed that proliferating neoblasts or new neurons were found in the penumbra area of the ischemic cortex after a stroke, some of which appeared to migrate 30 days after the stroke [51,58]. A definitive conclusion about endogenous poststroke neurogenesis is that although the proliferation period of SVZ cells appears to be short and only a few can eventually become mature neurons, this process is essential for stroke recovery [59]. The balance between the quiescence, activation, and proliferation of NSCs after a stroke determines not only the rate of neurogenesis but also the maintenance of the stem cell bank and the neurogenic capacity of the brain. This transition between the quiescent and proliferative states of NSCs is reversible and tightly regulated by multiple signaling pathways [60].

The Notch signaling pathway is a key modulator that determines the quiescent, proliferative, or differentiated states of NSCs and plays a vital role in their ability to maintain their undifferentiated state and self-renew [61]. Notch receptors are transmembrane, single-channel, heterodimeric proteins that undergo conformational changes by interacting with Notch ligands in adjacent cells to release the Notch intracellular domain (NICD). The NICD then transfers to the nucleus, which then combines with the recombination signal sequence binding protein Jκ (RBP-Jκ), a transcriptional repressor, to form a complex [62]. The NICD-RBP-Jκ complex functions as a transcriptional activator or inducer of transcription factor expression, causing the expression of various target genes, such as Hes family BHLH transcription factors 1 and 5 (Hes1 and Hes5), NeuroD, Mash1, Neurogenin1 (Ngn1), and Neurogenin2 (Ngn2) [63], and thus influencing the activation, proliferation, and differentiation of NSCs [64]. Hes genes are expressed in the SVZ, and Hes1 protein expression level oscillates in active NSCs and drives the cyclic expression of its target gene, Achaetescute homolog 1 (Ascl1), which activates cell proliferation [65].

The levels of Notch signaling and its downstream transcriptional target, Hes1, have been shown to increase in the SVZs of rats that underwent MCAO at 4 and 24 h after ischemia [66]. The inhibition of Notch1 signaling reduces the proliferation of SVZ cells in mice [67]. In contrast, an increase in the number of both proliferative cells in the SVZ and new cells expressing the immature neuronal marker Hu was observed in the rat cerebral cortex following the administration of the Notch ligand [68]. The Notch signaling pathway plays a role in the proliferation of SVZ progenitor cells in neurosphere cultures from adult rats [69]. Moreover, enhanced proliferation of NPCs in aged, ischemia-induced rats via the activation of the Notch1 signaling pathway resulted in a reduced infarct volume and improved motor deficits [70]. These findings suggest that Notch signaling mediates neuronal proliferation and adult neurogenesis in the SVZ under physiological and cerebral ischemic conditions. Targeting and modulating the Notch signaling pathway is feasible for improving neurogenesis after ischemic injury.

Shh is a pleiotropic signaling protein that modulates the activation, proliferation, and migration of NSCs [71]. The Shh signaling pathway increases the systemic division of NSCs to regulate self-renewal, which is crucial for behavioral recovery after a stroke [72,73]. On day 7 post ischemia, Shh protein levels increased significantly in mature neurons in the hippocampal region, especially in the CA3 and hilar regions [74]. In the adult hippocampus, the overexpression of Shh in neural progenitor cells within the DG via adenoviral transcription can increase cell proliferation after a stroke. In contrast, when the Shh signaling inhibitor cyclopamine was injected into the lateral ventricle and adult hippocampus, the proliferation of granule cells in the DG decreased [75,76,77]. These results suggest that Shh expression is upregulated in neurons after a stroke and that its inhibition reduces proliferation and neuronal neurogenesis.

The PI3K-Akt pathway also plays a critical role in the mechanism that regulates NSC activation and proliferation. FOXO3 is a key transcription factor that maintains the quiescent state of NSCs. FOXO3 and ASCL1 share several transcriptional target genes. FOXO3 competitively suppresses the expression of the Ascl1 target genes associated with the cell cycle to induce the resting state in NSCs [78]. The downstream mechanisms of AKT signaling that promote the activation or proliferation of NSCs include mTOR activation and FOXO3 phosphorylation and inactivation. The AKT-mTOR1 signaling cascade is an effective inducer of NSC activation and is usually considered the signaling integrator of nerves [79,80]. Several growth factors, including insulin family members and the MFGE8/integrin/ILK pathway, activate or suppress the AKT-mTOR1 signaling cascade to regulate NSC proliferation [81]. Mfge8 is secreted by the NSCs and astrocytes located in the SGZ and can bind to Itgb and activate phosphatase and tensin homolog, a dominant negative regulatory factor for PI3K activation, thus suppressing Akt-mediated mTOR activation. Hence, Mfge8 inhibits Akt-mediated mTOR activation, which is necessary for PI3K-Akt activation to induce NSC proliferation [82,83,84].

### 2.2. Migration

It has been well documented that after a stroke, dividing neoblasts migrate from the SVZ into the ipsilateral striatum and periinfarct area [85]. The migration of NSCs to areas of CNS injury and neurodegeneration is a critical step in the cell-mediated restoration of homeostasis in areas of injury [16,86,87,88]. During migration, these neoblasts may be remodeled and expressed as polysialylated neural cell adhesion molecules and doublecortin (DCX) [89,90].

Tropism is the natural tendency of NSCs to home in on the site of injury, in part through chemokine receptors on the NSCs [91]. The chemokine, SDF-1, and its corresponding receptor, CXCR4, are part of the most prevalent mechanisms that regulate migration. After an ischemic stroke, it was discovered that the SDF-1 receptor, CXCR4, was expressed in neural progenitors and stroke-generated neuroblasts and that the expression of SDF-1 was increased in reactive astrocytes and activated microglia in the injured area and stroke hemisphere [92]. Cortical interneurons and hippocampal dentate granule cells are two examples of cells influenced by the chemokine, SDF-1, and its corresponding receptor, CXCR-4, to migrate in a certain direction [93]. However, the inhibition of CXCR4 expression greatly attenuates neuroblast migration, suggesting that SDF-1/CXCR-4 signaling promotes the migration of neuroblasts to injured areas [20,92]. SDF-1 binding to CXCR4 can activate various signaling pathways, such as p38 MAPK, PI3K/Akt, c-Jun N-terminal kinase (JNK), and ERK1/2, to enhance stem cell migration [94]. The brain expresses osteopontin, a constitutive phosphoglycoprotein that is significant for tissue homeostasis and may exert therapeutic effects by upregulating CXCR4 expression and stimulating the migration of NSCs [95]. In addition, CC motif chemokine ligand 2 (CCL2) is one of the most popular chemokines expressed in ischemia. Studies have shown that the migration of NSCs to the infarct area and the subsequent neural repair are caused by an enhanced interaction between CCL2 and the CCL2 receptor (CCR2) [96,97].

Monocyte chemoattractant protein-1 (MCP-1) is another essential factor involved in neuroblast migration after a stroke. After a stroke, MCP-1 is upregulated in reactive astrocytes and microglia in the cortex and striatum, and the MCP-1 receptor, CCR2, is expressed in new neuroblasts. Moreover, various extracellular matrix (ECM) proteins are associated with neuroblast migration. Matrix metalloproteinases (MMPs), a family of proteases involved in ECM remodeling, are upregulated and participate in neuroblast migration after brain damage [98]. In mice that had strokes, MMP-9 in the SVZ and striatum colocalized with the neuroblast marker DCX [99]. The application of an MMP inhibitor significantly inhibited the migration of neuroblasts. In another mouse model of stroke, MMP-3 was expressed in migrating, DCX-positive neuroblasts. Endogenous MMP-3 and MMP-9 were found to promote chemokine migration using an in vitro assay system and specific siRNAs [100].

### 2.3. Differentiation

Neural function recovery after brain injury is marked by the completion of network reconstruction [101]. The functional differentiation of neurons is an anatomical basis for the reconstruction of neural networks in the brain [102]. The differentiation of NSCs is regulated by multiple signal interactions and is influenced by growth factors, cytokines, adhesion molecules, the extracellular matrix, and the cell microenvironment after a stroke [103].

Recent research suggests that FGF2 is an important regulatory factor for neurogenesis in the brain, and the injection of FGF2 into the lateral ventricle can increase the number of new cells formed in the hippocampal tissue of the adult brain [104]. Furthermore, conditional *FGFR1*-null mice showed neuronal progenitor cell proliferation and disturbances in new neuron generation in the DG region [105]. FGF-2 treatment enhances cell proliferation in the SVZ of neonatal rats with bilateral common carotid artery occlusion and accelerates the differentiation of these cells into neurons, astrocytes, and oligodendrocytes [106]. IGF-1 is a multifunctional promitotic factor that functions during development in the adult brain [107]. IGF-1 can directly stimulate hippocampal progenitor proliferation in the adult brain via MAPK kinase [108]. In parallel with promoting adult neurogenesis, IGF-1 suppresses BMP signal transduction to promote the differentiation of adult hippocampal progenitor cells into oligodendroglial cells in vitro and in vivo [109]. Vascular endothelial growth factor (VEGF) exhibits potent neurogenic activity after a stroke [110]. VEGF-A and VEGF receptor-1 were upregulated in the SVZ after a stroke [111]. A short-term VEGF-A injection into the ventricles improves cell proliferation in the SVZ and DG 28 days post stroke and is significant for functional recovery after a stroke by increasing angiogenesis and neurogenesis [24]. VEGF can directly promote the mitosis of neuron progenitor cells through an Flk-1-dependent mechanism [112]. In addition, VEGF-A enhances the differentiation of neuroglial progenitor cells into astrocytes in the SVZ. BDNF belongs to the neurotrophic factor family, which increases neurogenesis and the migration of neural progenitor cells from the SVZ, thus improving the recovery of sensorimotor function after a stroke [113]. The signal transduction of BDNF and its TrkB receptor can promote neuronal survival, dendritic arborization, and synapse formation to enhance neurogenesis [114]. BDNF also promotes gamma-aminobutyric acid release, which is important for promoting NSC differentiation and synapse formation [115].

Recent evidence has implicated the Wnt/β-catenin pathway in the proliferation and differentiation of NSCs in the adult brain and ischemic mouse models. Wnt proteins are primarily secreted by NSCs and astrocytes in neurogenic niches. The Wnt/β-catenin and Wnt/planar cell polarity pathways are the main pathways involved in NSC differentiation [116]. The genes, which code for Wnt (especially Wnt*1* and Wnt*3*), have been shown to be upregulated in late ischemic strokes and are thus involved in recovery after ischemic injury [117]. Wnt3 is highly expressed in hippocampal DG cells and can bind to and activate the transmembrane frizzled and low-density lipoprotein receptor-related protein 5/6 receptors in target cells. Subsequently, the activated receptor complex inhibits the activation of glycogen synthase kinase-3 (GSK-3β), stabilizing β-catenin within the cell, translocating it to the nucleus, and combining it with the lymphoid enhancer factor/T-cell factor (TCF). While Wnt ligands are lacking, GSK-3β is activated, leading to the intracellular degradation of β-catenin and the promotion of target gene transcription. The target genes that are associated with neurogenesis and modulated by the Wnt signaling pathway include paired box protein 6 (Pax6), distal-less homeobox 2, and octamer-binding transcription factor 4 [63]. It has been shown that after cerebral ischemia, Wnt3a mainly enhances the expression levels of β-catenin and TCF-4 and the downstream activation of the transcription factors Pax6 and Neurogenin2 in SD rats [118]. Pax6 is expressed in the SVZ and OB and has been shown to regulate the proliferation and differentiation of NSCs in vivo and in vitro [119,120,121,122]. After inducing focal cerebral ischemia in mice using a local injection of a lentivirus expressing Wnt3a-HA into the striatum or SVZ region, a dramatic increase in the number of differentiated BrdU-positive cells in the striatum into mature and immature neurons in the SVZ was observed [123]. Moreover, after 7 days of intranasal treatment with Wnt3a, BDNF expression levels were upregulated in adult mice subjected to MCAO/R [124].

## 3. Effects and Mechanism of Traditional Chinese Medicine (TCM) in Promoting NSCs Involved in Neurogenesis after an Ischemic Stroke

NSCs have been shown to be a potential source of replacement for degenerated neurons in CNS diseases. Using small molecules to induce endogenous NSC neurogenesis is a potential approach for generating the desired cell types in large numbers. Thus, the effects of TCM on NSCs have excellent prospects for application. It plays a significant role in promoting the activation, proliferation, migration, differentiation, repair of neuronal loss, and functional injury in NSCs because of its multicomponent and multitarget characteristics (Graphical abstract, Table 1 and Table 2).

*Momordica charantia*, sometimes referred to as bitter melon, is a traditional fruit widely used in the supplementary treatment of cardiovascular disease and diabetes. *M. charantia* polysaccharides (MCPs) are important bioactive components with hypoglycemic, cholesterol-reducing, antioxidant, and anti-obesity properties. MCPs have been demonstrated to improve NSC proliferation in the SVZ and SGZ and restore memory and learning ability in MCAO rats [125]. In addition, it promoted C17.2 cell proliferation in response to OGD injury. The potential mechanism relies on the upregulation of SIRT1 activity and cytoplasmic β-catenin deacetylation, which promote β-catenin nuclear translocation to induce NSC proliferation [125].

Pseudoginsenoside-F11 (PF11) is a saponin extracted from the leaves of *Panax pseudoginseng* ssp. In tMCAO mice, PF11 significantly reduced hippocampal atrophy, cognitive impairment, sensory dysfunction, infarction, and mortality. It also promoted neuroblast migration and newborn neuron survival in the ipsilateral striatum and DG, mediated mainly through the activation of the BDNF/TrkB pathway, thereby improving long-term nerve damage and enhancing neurogenesis after a stroke, indicating its potential role in the convalescent treatment of ischemic strokes [126].

Ginsenoside, the primary active ingredient in ginseng, is widely used to treat acute ischemic strokes and ameliorates mitochondrial dysfunction by inhibiting oxidative stress, glutamate neurotoxicity, and apoptosis. Ginsenosides also support the differentiation and proliferation of NSCs. Ginsenoside can increase the optical density and area density, the number of cells positive for nestin/BrdU, nestin/vimentin, and nestin/tuj-1, and the expression levels of BrdU, tuj-1, and vimentin, suggesting that it may promote NSC proliferation and differentiation in neurons and astrocytes. In addition, ginsenosides can activate the expression of HIF-1α and VEGF proteins, indicating that neurogenesis is related to HIF-1α-VEGF pathway activation [127]. A recent study revealed that ginsenoside Rb1 promotes long-term motor function recovery and improves cortical axon regeneration after a stroke. CREB, a vital transcription factor for cell growth and development, is a phosphorylation substrate in numerous signaling pathways, including cAMP, calmodulin, and NMDA receptors [128]. Cerebral ischemia induces a massive release of glutamic acid, which activates the CREB pathway by interacting with NMDA receptors, thus inducing the expression of Bcl-2 and BDNF [129]. Research suggests that cAMP-mediated CREB phosphorylation promotes NSC proliferation and neuronal survival in the adult hippocampus. Moreover, the spontaneous knockdown of CREB in mouse hippocampal cells can impair the differentiation and maturation of newly formed hippocampal granule cells [130]. CREB signaling is essential for regulating the survival, migration, and morphological differentiation of neuroblastomas in the SVZ [128]. Interestingly, the deletion of CREB results in increased Pax6 expression, indicating that the effects of CREB signal transduction on the survival of immature neurons in the RMS may be regulated by Pax6 [131]. After 14 days of intervention, ginsenoside Rb1 increased GAP43 and BDA expression in the ipsilateral and contralateral cortices of mice with distal middle cerebral artery occlusion and improved motor function, which may have been due to the regulation of the cAMP/PKA/CREB signaling pathway [132].

Astragali radix (AR), a widely used herb in TCM, is the main ingredient of the Buyang Huanwu decoction used for the treatment of strokes. Many active ingredients extracted and isolated from AR have shown significant neuroprotective effects in a variety of experimental animal models of stroke, of which Astragaloside VI (AS-VI) has been found to promote neurogenesis. Treatment with AS-VI (2 μg/kg) for 7 days promoted spatial learning, memory, and motor function in transiently ischemic rats by inducing neurogenesis and astrogenesis in the DG, SVZ, and cortex. In addition, it enhanced the self-renewal and proliferation of NSCs in vitro. Adult NPCs express the EGF receptor (EGFR), which is upregulated under ischemic conditions to enhance their susceptibility to EGF or other EGFR ligands in response to ischemia [126]. EGF intervention promotes neuronal differentiation in the corpus striatum after the onset of ischemia in newly formed parvalbumin neurons [133]. AS-VI intervention upregulates nestin, p-EGFR, and p-MAPK protein expression and neurosphere size; however, combined treatment with the EGF receptor inhibitor, gefitinib, and ERK inhibitor, PD98059, reverses this effect [134]. AS-VI may be a therapeutic candidate for adult neurogenesis and brain repair via targeting the EGFR/MAPK signaling pathway after stroke treatment.

Several clinical and laboratory-based studies have shown that after ischemic strokes, AS-VI, another major active component of *Astragali radix*, confers neuroprotective effects to patients. Recent studies have indicated that in a photochemical ischemia model established in C57BL/6 mice, AS-VI intervention promoted hippocampal neurogenesis, with a significant increase in apical dendrite length and spine density after a stroke [126,135]. In addition, AS-VI reversed neuronal apoptosis, neurogenesis, and cognitive dysfunction in interleukin (IL)-17 knock-out mice. Further studies confirmed that the effects of AS-VI in promoting brain repair and improving stroke-induced cognitive impairment are mainly mediated by the downregulation of IL-17 expression through the Wnt and Akt/GSK-3β pathway signaling pathways.

Astragalus polysaccharide (APS) is an active ingredient with antitumor, antiviral, anti-inflammatory, and antioxidant effects [23]. Furthermore, the astragalus extract has been reported to have a protective effect against hippocampal neuronal damage caused by intermittent hypoxia in rats. APS pretreatment attenuates hypoxia-induced hippocampal NSC injury by increasing cell viability and reducing apoptosis. The upregulation of miR-138 expression following APS pretreatment was reversed by an miR-138 inhibitor. In addition, APS inhibited the JNK and p38MAPK pathways via miR-138. The neuroprotective effects of APS against hypoxia-induced NSC injury may be achieved by upregulating miR-138 expression and inhibiting the JNK and p38MAPK pathways.

Salvianolic acid A (SAA), one of the main water-soluble, active ingredients of *Salvia miltiorrhiza* Bge., has a variety of pharmacological effects, including anti-apoptosis, antioxidative stress, antiinflammation, and neurovascular protection, and is widely used in cardiovascular and cerebrovascular diseases. The long-term administration of SAA significantly reduced infarction volume, vascular embolism, neurological deficits, and pathological damage in the hippocampus and striatum. In addition, SAA significantly increased the proliferation and migration of NSPCs, promoted their differentiation into neurons, enhanced axonal regeneration, and reduced the apoptosis of neurons around the ipsilateral SVZ region, which led to the restoration of neural density in the ischemic striatum and the reconstruction of neural circuits. The underlying mechanism is related to the activation of the Wnt3a/GSK3β/β-catenin signaling pathway [136].

Tanshinone IIA (TIIA), a major lipophilic component, is extracted from *Salvia miltiorrhiza* Bge. (Danshen). TIIA can rescue impaired neurons after acute ischemic injury through various targets, such as attenuating oxidative stress, platelet aggregation, and blood–brain barrier disruption. TIIA activates MAPK42/44 and downstream transcription factor CREB in PC12 cells. Endogenous nerve growth factors (NGF) promote neurogenesis after a stroke. The intranasal injection of NGF has been shown to improve the survival of freshly generated neurons in the ipsilateral striatum and SVZ of rats that underwent unilateral MCAO without affecting cell proliferation [137]. TIIA upregulated NGF and BDNF expression, and the differentiation effects were partially attenuated by MEK inhibitors and antagonists of NGF and BDNF receptors, suggesting that the BDNF and NGF signals mediated by MAPK42/44 are involved in the prodifferentiation effects of TIIA. Caveolin-1 (CAV-1), the major functional protein in membrane caveolae, plays an essential role in the endocytosis of exogenous substances. Transmembrane transport of TIIA was enhanced by activating CAV-1, which initiated differentiation. The inhibition of CAV1 expression further suppresses the prodifferentiation effects of TIIA, suggesting that TIIA may partially exert neuroprotective effects in a caveolae-dependent manner through BDNF and NGF signaling mediated by MAPK42/44 [133].

Artesunate (ART) is a derivative of artemisinin that is highly effective for malaria treatment. A previous study showed that ART ameliorated blood–brain barrier damage in mice with subarachnoid hemorrhage, mainly by mediating the S1pR/PI3K signaling pathway. Moreover, studies have revealed that ART reduces ischemic brain volume and white matter lesions in MCAO mice and increases the proportion of BrdU-positive endogenous NSPCs in the ipsilateral SVZ and periinfarct cortex. However, the neurorestorative effects of ART were offset by FOXO3a overexpression, suggesting that ART can ameliorate ischemia/reperfusion damage by promoting endogenous NSPC neurogenesis and proliferation via the FOXO3a/p27Kip1 pathway [138].

Osthole (Ost), a natural coumarin derivative extracted from *Cnidium monnieri* (L.) Cusson, exhibits anti-inflammatory, antiapoptotic, antioxidative, and neurotrophic properties. Previous studies have demonstrated the role of Ost in promoting the proliferation of NSCs in vitro and improving neurogenesis in the hippocampus of APP/PS-1 double transgenic mice [139]. In addition, it promoted the proliferation of endogenous NSCs, improved neuronal restoration in regions of brain injury and the hippocampal DG and CA3, and improved learning and memory function in MBI mice. Ost upregulated *Notch1* and *Hes1* gene expression and NICD and Hes1 protein expression, which were blocked by the c-secretase inhibitor DAPT, suggesting that the neuroprotective effects of Ost are partly involved in the activation of the Notch signaling pathway [137].

Crocin, a series of ester glycosides formed by a combination of crocin acid and various sugars, is obtained from a perennial iris crocus (*Crocus sativus* L.) with a dry stigma. Crocin inhibits the Bax/Bcl-2 ratio in endogenous NSCs, reduces inflammatory factor release, and enhances Notch1 expression after cerebral ischemia reperfusion in the rat brain. Furthermore, the neuroprotective effects of crocin on NSC proliferation and migration were demonstrated by mediating the Notch signaling pathway in a hypoglycemic/reoxygenation model [140].

Ellagic acid (EA) is an antioxidant compound derived from pomegranate and is used to treat muscle spasms and neuropathic aches in TCM. In addition, EA has been recommended as a potential treatment for various CNS disorders, such as strokes and dementia. The administration of EA alleviated infarct volume and improved neurological function and nestin protein levels in rat ischemic penumbra. Increased cell proliferation and the upregulation of the *β-catenin* and *cyclin D1* genes of NSCs were also observed in primary cultured NSCs, suggesting that EA can ameliorate brain injury and promote the proliferation of NSCs through the Wnt/β-catenin signaling pathway [141].

## 4. Discussion and Conclusions

Stroke is a leading cause of death in China with an increasing prevalence. Globally, China has the highest estimated lifetime risk of stroke at 25 years of age and beyond. The risk factors, including the aging population, the prevalence of diabetes, obesity, and hypertension, and physical inactivity, demonstrated upward trends in the 2010s. Global Burden of Disease data have reported that the age-standardized incidence and mortality rate declined from 1990 to 2019; however, a plateau or increasing trend in the incidence and mortality rates of stroke in China were noted [142].

To date, the treatment of ischemic stroke continues to be a daunting task due to the lack of effective treatment options. Neurogenesis is critical for brain development and damage repair. As the CNS has a limited repair capacity, it is clinically valuable to seek alternative methods to promote recovery after nerve injury [14]. Cellular therapy, especially stem cell therapy, is an emerging field of research in neurological disorders and has long been a promising innovative alternative to acute thrombolysis [33] and chronic rehabilitation [143,144] for many neurological diseases or injuries. Recent studies have shown that endogenous NSCs in pathological conditions such as strokes can be modulated to reverse CNS functional impairment. Overall, this endogenous treatment approach can be achieved by modulating neuroinflammatory responses, targeting neuronal replacement, improving intracerebral nutritional support, remodeling neural circuits, restoring neurological function, and modulating the paracrine signaling of nerve growth factors [54]. However, the therapeutic strategies aimed at promoting endogenous neurogenesis face numerous challenges. First, there are a series of dramatic changes in the cellular microenvironment due to damage to the blood–brain barrier, excitotoxicity, neuroinflammation, acidosis, and the production of reactive oxygen species after stroke, which affect endogenous neurogenic processes [50]. The mechanisms underlying the activation, proliferation, migration, differentiation, and integration of NSCs after a stroke are complex and require further investigation. Additionally, the functional recovery process and structural repair mechanisms of the post-stroke neural network microenvironment must be explained in detail. Second, the number and distribution of endogenous NSCs in the adult brain are limited. A stroke results in a series of cellular microenvironmental effects that usually cause endogenous NSCs to differentiate into glial cells rather than neurons, and most NSCs do not reach damaged cortical areas for functioning [145,146]. Therefore, therapeutic strategies that rely solely on the promotion of endogenous NSC activation are inadequate for promoting post-ischemic neurogenesis and repair. The development of an effective and safe endogenous NSC strategy in combination with exogenous stem cell implantation has great translational significance.

For decades, TCM has been a popular research topic for the prevention and treatment of CNS diseases, and its safety has been demonstrated through long-term clinical use. Chinese medicine has the advantages of holistic regulation and the comprehensive treatment of incurable neurological diseases, such as strokes. It is well known that the pathological environment generated after cerebral ischemic injury is not conducive to endogenous neurogenesis and repair. Chinese medicine may be an effective therapeutic option to promote the neuroprotection, antiapoptotic differentiation, and proliferation of NSCs, improve the microenvironment in the brain, and reduce neuroinflammation after a stroke. Several meta-analyses have shown that Chinese medicine can enhance clinical recovery and significantly improve neurological deficits in stroke patients, thus improving their quality of life and overall therapeutic efficacy [147]. In this review, we summarized Chinese medicines that regulate endogenous neurogenesis after a stroke and their related mechanisms (Table 1 and Table 2). However, we also noted that only some of these studies were of a high quality, the majority of the data were generated in cell culture models with short-term outcomes, supportive, robust in vivo experiments were limited, and human clinical trials and epidemiologic data were relatively inadequate, reminding researchers conducting follow-up studies to pay attention to these urgent situations. Moreover, problems with repair/regeneration in the CNS are not unique; instead, they are attributes of most terminally differentiated organs and tissues. It is worth studying whether Chinese herbal medicines exert repair/regenerative abilities in these organs and tissues. Owing to the diversity of the active ingredients of Chinese herbal medicines, the correspondence between herbs, ingredients, targets, and diseases is not easily clarified, resulting in an insufficient depth of relevant research. Therefore, the application of technologies such as high-throughput screening, small-molecule probes, label-free detection, and target identification and validation should be strengthened to thoroughly analyze network relationships and integrate regulatory processes between the signaling pathways regulated by Chinese medicine. Since no single compound will effectively treat or cure stroke, a means of integrating the use of individual substances or herbs to produce holistic treatment and illustrate the mechanism is still a challenge.

**Table 1:** Traditional Chinese medicine monomer and its pharmacology on endogenous neural regeneration in vivo.

Source	Classification	Species	Dosage	Treatment Route	Neurogenic Region	Model	Mechanism	Phenotype	Reference
*Momordica charantia* (Ku Gua)	M.charantia polysaccharides	Rat	200 mg/kg	Intragastric administration	SVZ/SGZ	MCAO	SIRT1, cytoplasmic, β-catenin, deacetylation	Rescue the memory and learning abilities of rats; enhance NSC proliferation	[125,148]
*Panax pseudoginseng* subsp (San Qi)	Pseudoginsenoside-F11	Mice	16, 32 mg/kg	Orally treated	DG	tMCAO	pro-BDNF, TrkB-T; ↑m-BDNF, TrkB-FL, p-AkT, p-CREB	Reduce brain infarction and brain edema; attenuate the mortality, sensorimotor dysfunction, cognitive impairment, and hippocampal atrophy	[126]
*ginseng* (Ren Shen)	Ginsenoside Rb1	Mice	50 mg/kg	Intraperitoneal injection	SVZ/SGZ	dMCAO	↑cAMP, ↑PKA, ↑p-CREB	Improve functional recovery; stimulate axonal regeneration and brain repair	[132]
*Radix Astragali* (Huang Qi)	Astragaloside VI	Rat	2 μg/kg	Intravenous injection	SVZ/DG	MCAO,	Nestin, p-EGFR, p-MAPK	Promote spatial learning and memory; improve impaired motor function	[134]
*Radix Astragali* (Huang Qi)	Astragaloside IV	Mice	200 mg/kg	Intravenous injection	Hippocampus	IL-17 KO mice, Photochemical brain ischemia model	p-Akt, p-GSK-3β, Wnt2, β-catenin, Nestin, IL-17, Wnt	Ameliorate stroke-induced cognitive deficits; repair spines of apical dendrites in the hippocampus; stimulate hippocampal neurogenesis; inhibite neural apoptosis; relieve anxiety after stroke	[135,149]
*Salvia miltiorrhiza* Bge (Dan Shen)	Salvianolic acid A	Rat	10 mg/kg	Intragastric administration	SVZ/ Hippocampus	Electrocoagulation-induced autologous thrombus stroke model	Wnt3a, p-GSK3β/GSK3β, β-catenin, TCF-4	Decrease infarction volume and vascular embolism; ameliorate pathological injury; promote NSPC proliferation, migration and differentiation; enhance axonal regeneration and diminish neuronal apoptosis	[136]
artemisinin (Qing Hao Su)	Artesunate	Mice	150 mg/kg	Intraperitoneal administration	SVZ	MCAO	Penumbra damage, white matter injury, FOXO3a, p27Kip1; DCX	Rescue ischemia damage; alleviate white matter injury; promote functional recovery; promote neurogenesis and proliferation of endogenous NSPCs	[138]
*Cnidium monnieri* (L.) (She Chuang Zi)	Osthole	Mice	30 mg/kg	Intraperitoneal administration	SVZ/SGZ/DG/ CA3	Model of stab wound injury is created to mimic the neuroendoscopy procedure	Notch-1, Hes-1, Nestin, NICD	Improve learning and memory function; promote the proliferation of endogenous NSCs; improve neuronal restoration; increase the number of neurons in the regions of brain injury	[137]
*Crocus sativus* L. (Fan Hong Hua)	Crocin	Rat	10, 50 mg/kg	Intragastric administration	SVZ/DG	MCAO/R	Bax/bcl-2; Notch1	Inhibit the release of inflammatory factors; reduce the apoptosis of nerve cells	[140]
*Pomegranates* (Shi Liu)	Ellagic acid	Rat	10, 30, 90 mg/kg	Intragastric administration	SVZ/SGZ	Photothrombotic nerve injury model	nestin, β-catenin, Cyclin D1	Improve the rats’ nerve-related abilities; remedy infarct volumes and morphological changes in the brain	[141]

**Table 2:** Traditional Chinese medicine monomer and its pharmacology on endogenous neural regeneration in vitro.

Source	Classification	Species	Dosage	Model	Mechanism	Phenotype	Reference
*Momordica charantia* (Ku Gua)	M.charantia polysaccharides	C17.2 cells, primary cortical neural stem cells	5 μg/mL	OGD, IRI	SIRT1, cytoplasmic, β-catenin, deacetylation	Change intracellular redox state; stimulate the proliferation	[125,148]
*Panax pseudoginseng* subsp (San Qi)	Pseudoginsenoside-F11	Primary cultured NSCs	100 μm	OGD/R	pro-BDNF, TrkB-T; ↑m-BDNF, TrkB-FL, p-AkT, p-CREB	Promote proliferation and differentiation	[126]
*ginseng* (Ren Shen)	Ginsenoside	Primary cultured NSCs	1 μg/mL	OGD/R	HIF-1α, VEGF	Maintain NSC replication; promote NSC proliferation; promote NSC differentiation into neurons and astrocytes	[127]
*Radix Astragali* (Huang Qi)	Astragaloside VI	C17.2 cells or primary cultured NSCs	10, 100 nM	DMEM/F12 media deprived of EGF or normal DMEM/F12 media stimulated for 2 h	Nestin, p-EGFR, p-MAPK	Enhance NSCs self-renewal and proliferation without affecting NSCs	[134]
*Radix Astragali* (Huang Qi)	Astragaloside IV	Primary cultured NSCs	10 nM, 100 nM, 20 μmM	/	p-Akt, p-GSK-3β, Wnt2, β-catenin, Nestin, IL-17, Wnt	Promote hippocampal neurogenesis and NSC proliferation	[135,149]
*Salvia miltiorrhiza* Bge *(Dan Shen)*	Tanshinone II A	C17.2 cells, primary culture of embryonic cortical NSCs or PC12 cells	0.1–3 μM	TIIA stimulated for 7 d	p-MAPK42/44, p-CREB, BDNF, NGF, GAP-43	Promote neuronal differentiation; facilitate endocytosis and transportation across the cell membrane	[133]
*Crocus sativus* L. (Fan Hong Hua)	Crocin	NSCs	10, 50 μM		Bax/bcl-2; Notch1	Promote cell proliferation; increase cell migration; inhibit cell apoptosis; and promote neural regeneration	[140]
*Pomegranates* (Shi Liu)	Ellagic acid	Primary cultured NSCs	1, 3, 9 μg/mL	OGD/R	nestin, β-catenin, Cyclin D1	Increase proliferation of NSCs	[141]

## Data Availability

Data sharing not applicable.
